# The relationships between resilience and child health behaviors in a national dataset

**DOI:** 10.1038/s41390-024-03664-9

**Published:** 2024-10-21

**Authors:** Ellen L. McMahon, Shelby Wallace, Lauren R. Samuels, William J. Heerman

**Affiliations:** 1 Vanderbilt University Medical Center, Department of Pediatrics, Nashville, TN, USA.; 2 Vanderbilt University Medical Center, Department of Biostatistics, Nashville, TN, USA.

## Abstract

**BACKGROUND:**

Resilience mechanisms at the individual, family, and environmental levels may improve health outcomes despite potentially harmful stress exposure partly through the practice of positive health behaviors.

**METHODS:**

We performed a secondary analysis of 2016–2021 National Survey of Children’s Health data to assess the relationships between three resilience domains – child, family, neighborhood – and six health behaviors using multiple regression models adjusted for the other resilience domain(s) and potential confounders.

**RESULTS:**

Analysis revealed significant associations between each resilience domain and multiple health behaviors in a total weighted analytic sample of 70,156,540 children. For each outcome, the odds of better health behaviors were highest with high resilience in all possible domains. For example, among children ages 0–5 years, the adjusted odds of having “good quality” vs. “poor quality” sleep for those with “high” resilience in all domains were 2.21 times higher (95% CI 1.78, 2.63) than for those with “low” resilience in all domains.

**CONCLUSIONS:**

This line of research may help to inform the design of resilience and health behavior promotion interventions by targeting multiple socio-ecological domains of influence to improve health and development outcomes in children exposed to experiences or sources of potential stress.

## INTRODUCTION

Health behaviors are responsible for a substantial burden of poor health outcomes across the lifespan, including mental health disorders, diabetes, cardiovascular disease, and cancer.^1-6^ In children, unhealthy patterns of diet and physical activity are associated with higher rates of obesity, diabetes, depression, and asthma.^7-10^ Prior studies have demonstrated associations between family health behaviors, such as eating meals as a family and reading together regularly, and child health and developmental outcomes, including nutritional health, body mass index, language development, literacy, and behavior.^11-17^ While general adherence to recommended health behavior guidelines is low throughout the United States (U.S.),^18,19^ among low-income and marginalized racial and ethnic populations there is a concordance of the lowest rates of engagement in healthy behaviors and the highest prevalence of poor health outcomes.^20-28^ Thus, characterizing the individual and contextual factors that influence health behaviors, especially early in the life course, may thus prove constructive for initiatives aimed at reducing health disparities.^24,27,29^

Resilience, defined as a person’s capacity to transform challenging or adverse experiences, sometimes described as sources of “toxic stress,” into manageable stress, is multifactorial, dynamic, and protective against many of the poor health outcomes that result from adversity.^30-32^ The concept of resilience as a mediator of health outcomes rejects the idea that unhealthy behaviors are the product of individual failures.^33,34^ Rather, health behaviors are influenced by larger social and structural factors,^21,27,35,36^ and resilience components can exist at multiple levels of a person’s social ecology, including at the individual, family, and community levels.^32,33,37-39^ Research has revealed an apparent reciprocal relationship between resilience and positive health behaviors, such that the two represent a positive feedback cycle contributing to health promotion.^40-46^

Despite a strong conceptual linkage and the existing literature in multiple adult populations,^32,40-46^ the associations between resilience and health behaviors have not been well described in children. Studies in adolescents are largely limited to those with high rates of adverse childhood experiences (ACEs), but have shown consistent associations between higher individual resilience and physical activity, healthy diet, better sleep, and lower rates of substance use.^31,47-49^ Studies in younger children are similarly limited to specific subpopulations, resilience domains, and health behaviors.^50,51^ To our knowledge, there has yet to be a study assessing the associations between multiple resilience domains and multiple health behaviors in a general population of children. This study thus aims to advance the field by describing the relationships between three socio-ecological levels of resilience and six child and family health behaviors in a nationally representative sample of children. Understanding these relationships may help to provide conceptual evidence for the inclusion of resilience promotion efforts in health behavior interventions and vice versa.

The purpose of this study was to describe the associations between three socio-ecological domains of resilience - child, family, and neighborhood - and the six health behaviors: child physical activity, child screen time, child sleep quality, frequency of family meals, and frequency of two household literacy-promoting behaviors (caregivers reading to and singing or storytelling to the child). We hypothesized that higher resilience in each of the measured domains would be associated with a greater likelihood of engaging in positive health behaviors; namely, more physical activity, less screen time, better sleep quality, more family meals, and more literacy-promoting behaviors.

## METHODS

### Survey design

We conducted a secondary analysis of data collected via the National Survey of Children’s Health (NSCH) between 2016 and 2021.^52^ The NSCH is an annual, cross-sectional survey of caregivers in households with at least one child under 18 years, conducted by the U.S. Census Bureau in order to assess the physical and mental health of children living in the U.S. The data and survey administration methods are publicly available and all data are nonidentifiable.^52^ This study was approved by the Vanderbilt University Medical Center Institutional Review Board.

The NSCH is conducted in English and Spanish and administered online and by mail. The Census Bureau identifies households in each state likely to have a child living in the home. These households are then screened to confirm eligibility and one index child per household is randomly selected as the survey subject. One of three survey forms, each targeting similar topics though with some variance in specific questions, is then administered based on the age of the child (0–5 years, 6–11 years, and 12–17 years).^53^ The respondent is a parent or caregiver in the home who has knowledge of the child’s health. Each survey is assigned a weight that includes adjustments for the base sampling weight, non-response, the selection of a single index child in a household, and demographic characteristics to obtain population-based estimates, as per the NSCH methodology guide. Results are thus representative of all non-institutionalized children ages 0–17 years living in the U.S. during the survey years whose caregivers speak English or Spanish. The weighted survey response rate was 40.7% in 2016, 37.4% in 2017, 43.1% in 2018, 42.4% in 2019, 42.4% in 2020, and 40.3% in 2021.

### Exposures: resilience domains

Resilience domains were measured by the NSCH using three separate scales: 1) child resilience, 2) family resilience, and 3) neighborhood resilience. Domains assessed differed by NSCH age-specific survey. All three domains were measured in ages 0–5 years. Family and neighborhood, but not child, resilience domains were measured in ages 6–17 years. Each domain scale consisted of four to five survey items (Appendix). We averaged responses across survey items within each domain to create continuous measures of resilience (range: 0–3, where 3 indicates the highest possible resilience within that domain) for each child at each age-applicable domain.

### Outcomes: health behaviors

Slightly different sets of health behaviors were measured by NSCH surveys in the 0–5 year age group and the 6–17 year age group. The six outcomes included in this analysis were: 1) average weekly frequency of child physical activity of at least 60 min (6–17 years only), 2) average daily hours of child screen time (both age groups), 3) child sleep quality (both age groups), 4) average weekly frequency of eating meals together as a family (both age groups), and two literacy-promoting behaviors (0–5 years only), 5) average weekly frequency of caregivers reading to the child and 6) average weekly frequency of caregivers singing or storytelling to the child. Screen time was measured as a five-level ordinal variable indicating the child’s average daily hours of screen time. This was then reverse-coded such that higher values represent less screen time for consistency with the other outcome measures in which higher values indicate better behavior outcomes. Sleep quality was measured via two questions, which we collapsed into a binary variable with possible outcomes of “good” or “poor” sleep quality, where “good sleep quality” indicates both a consistent bedtime and an average daily sleep amount within the recommended range for that child’s age according to the American Academy of Sleep Medicine’s guidelines.^54^ All other outcomes were collected as single questions using 4-point Likert scales. See Appendix for complete outcome measurement details.

### Covariates

Covariates included child age (years & months), child sex (male, female), NSCH-defined child race categories (White alone, Black or African American alone, Other), child ethnicity (Hispanic or Latino origin, Not Hispanic or Latino origin), survey year (2016-21), primary household language (English, Spanish, Other), highest level of education among household adults (Less than high school, High school, Some college or associate degree, College degree or higher), family poverty ratio (≤50–99%, 100–199%, 200–399%, ≥400%), and child special healthcare needs (yes, no). Child race/ethnicity was categorized as “Hispanic” if “Hispanic or Latino origin” was reported; and otherwise categorized as “Non-Hispanic White”, “Non-Hispanic Black”, or “Non-Hispanic Other.” Exposure to ACEs was measured via nine binary items (Appendix), then categorized by count into the following ranges for analysis: 0, 1–3, and 4–9 ACEs.

### Statistical analysis

Because slightly different sets of exposures and outcomes were measured in the 0–5 vs. 6–17-year-old surveys, we performed separate analyses by these two age groups. For each health behavior outcome within each age group, we fit a multiple regression model (binary logistic regression for sleep quality; ordinal logistic regression for all other outcomes), adjusting for all other age-applicable resilience domain(s) and covariates. In ages 0–5 years, we fit models for the five measured outcomes (screen time, sleep quality, family meals, reading, and singing/storytelling), adjusting for the three measured resilience domains (child, family, and neighborhood) and covariates. In ages 6–17 years, we fit models for the four measured outcomes (physical activity, screen time, sleep quality, and family meals), adjusting for the two measured resilience domains (family and neighborhood) and covariates. We then modeled each relationship with the addition of interaction terms (i.e., age category by family resilience and age category by neighborhood resilience) to assess for differences by age group within the 6–17 year cohort between “middle childhood” (ages 6–11 years) and “adolescence” (ages 12–17 years).

For each health behavior outcome within each age group, we report results via adjusted odds ratios (aOR) and predicted probabilities, both with 95% confidence intervals (CI), for a set of resilience profiles. Each resilience profile represents a different combination of “low resilience” (score of 1.5 on the corresponding resilience scale) and “high resilience” (score of 3.0 on the corresponding resilience scale) domains. The aORs compare the odds of reporting a “better” vs “worse” health behavior outcome for the specified profile compared to the profile with “low resilience” in all age-applicable domains. In their respective models, we defined the “better” health behavior outcomes as more frequent physical activity, less daily screen time, good as opposed to poor sleep quality, more frequent meals eaten together as a family, or more frequent literacy-promoting behaviors. To present results on an absolute rather than relative scale, we then give the predicted probabilities of reporting the “best” health behavior for each outcome by resilience profile. Given the reported differences in health behavior rates, we present these predicted probabilities for each race/ethnicity category; in each case, all other covariates are set to the median or modal value observed within each age group.^54,55^

All analyses were conducted following NSCH-provided methodology for combining and weighting multi-year survey data.^56^ Only surveys with complete outcome data were included in the analysis. Missing exposure and covariate data were multiply imputed via predictive mean matching on raw NSCH data and the following derived variables: resilience scores, sleep quality, screen time, number of ACEs, and race/ethnicity.^20^ All values with a missing value code or imputed by NSCH were converted to missing prior to imputation for consistency. Model-based estimates were derived from an overall model combining 20 imputed datasets using Rubin’s rules, and 95% CIs for estimates were produced via a parametric bootstrap with 10^4^ iterations.^21^ All analyses were conducted in R (version 4.2), with the “survey” and “mitools” packages.^24,52,57^

## RESULTS

### Baseline demographics

The publicly available 2016–2021 NSCH datasets contain 70,265 and 155,178 surveys from households with children aged 0–5 and 6–17 years, respectively.^58^ After removing surveys without complete outcome data from our dataset, the analytic samples for this study consisted of 68,119 surveys representing a weighted sample of 22,526,805 children aged 0–5 years and 150,139 surveys representing a weighted sample of 47,629,735 children aged 6–17 years. All results are based on the weighted samples using survey weights as assigned by the NSCH. Baseline demographics are presented in Table 1. The median (interquartile range) age was 3.0 years (1.0–4.0 years) in the 0–5 year age group and 12.0 years (9.0–14.0 years) in the 6–17 year age group. Categorical, parent-reported child race/ethnicity in ages 0–5 years was 24.0% Hispanic, 52.0% non-Hispanic White, 12% non-Hispanic Black, and 12.0% non-Hispanic other; and in ages 6–17 years was 26.0% Hispanic, 51.0% non-Hispanic White, 13.0% non-Hispanic Black, and 10.0% non-Hispanic other. In both age groups, 51.0% of children were male and 86.0% reported English as the primary household language. Most children aged 0–5 years (55.0%) lived with an adult with a college degree or higher vs. 48.0% of children aged 6–17 years.

### Exposure and outcome results

Table 2 shows resilience profile distribution by age group (see Supplementary Table 1 for detailed exposure and outcome results). In the 0–5 year age group, 31.5% of children had high resilience (score between 2.5–3) in all three domains and 0.1% had low resilience (score ≤1.5) in all three domains. In the 6–17 year age group, 31.2% of children had high resilience in both domains and 1.7% had low resilience in both domains.

### Adjusted associations between resilience and health behaviors

**Fig. 1:**
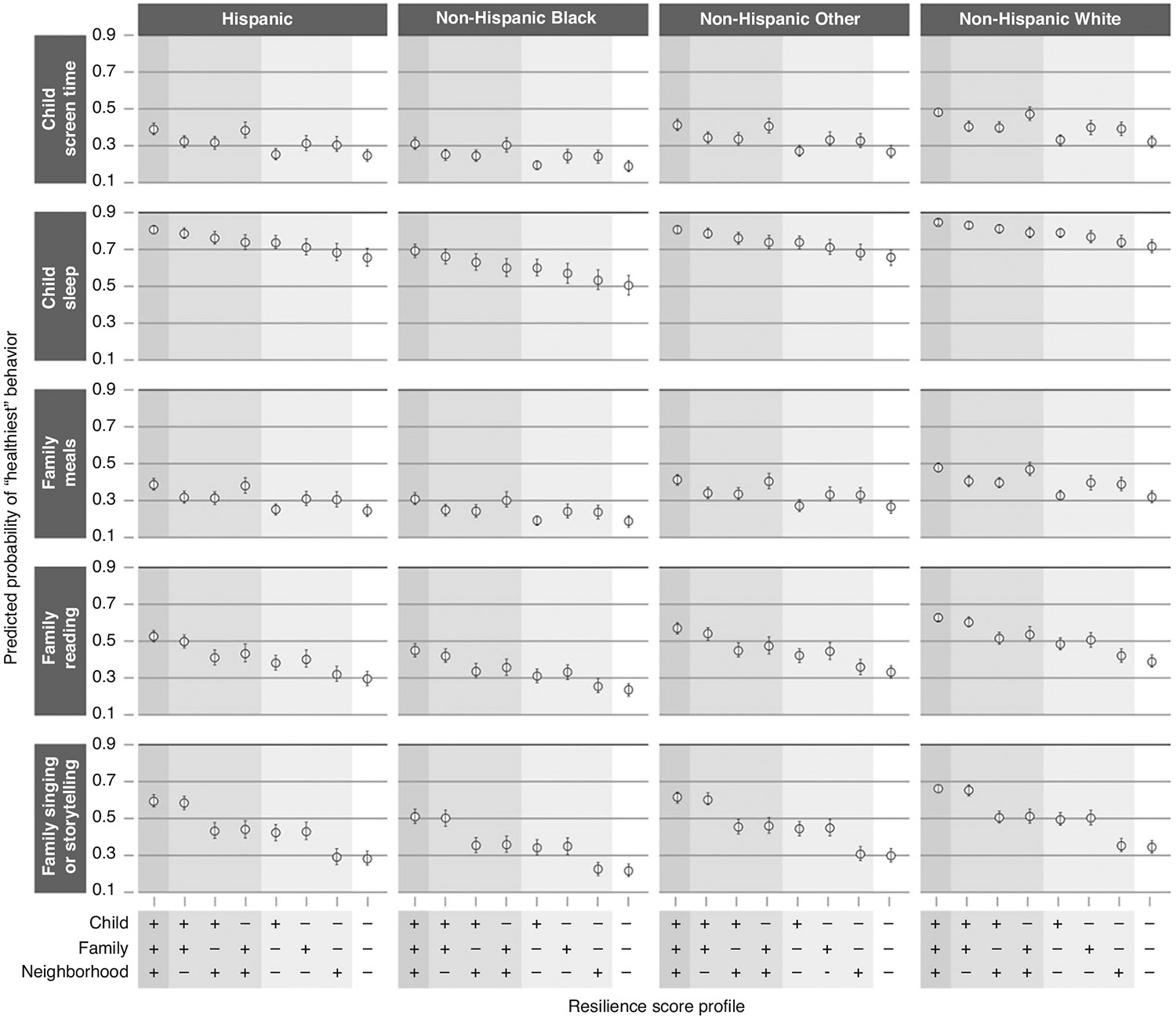
Predicted probabilities of “healthiest” behavior outcomes in 0–5 years. Results are displayed by race/ethnicity, indicated in column headings, and by health behavior outcome, indicated in headings on the left. Resilience profiles are designated along the x-axis at the bottom of the figure. For each domain (child, family, and neighborhood), “+” indicates “high resilience” (score of 3.0) and “−” indicates “low resilience” (score of 1.5) in that domain. Covariates for all resilience profiles are set to the median or modal value. For each health behavior outcome, the y-axis represents the predicted probability of reporting the “healthiest” behavior option, with values indicated by open circles and vertical error bars representing 95% confidence intervals.

**Fig. 2:**
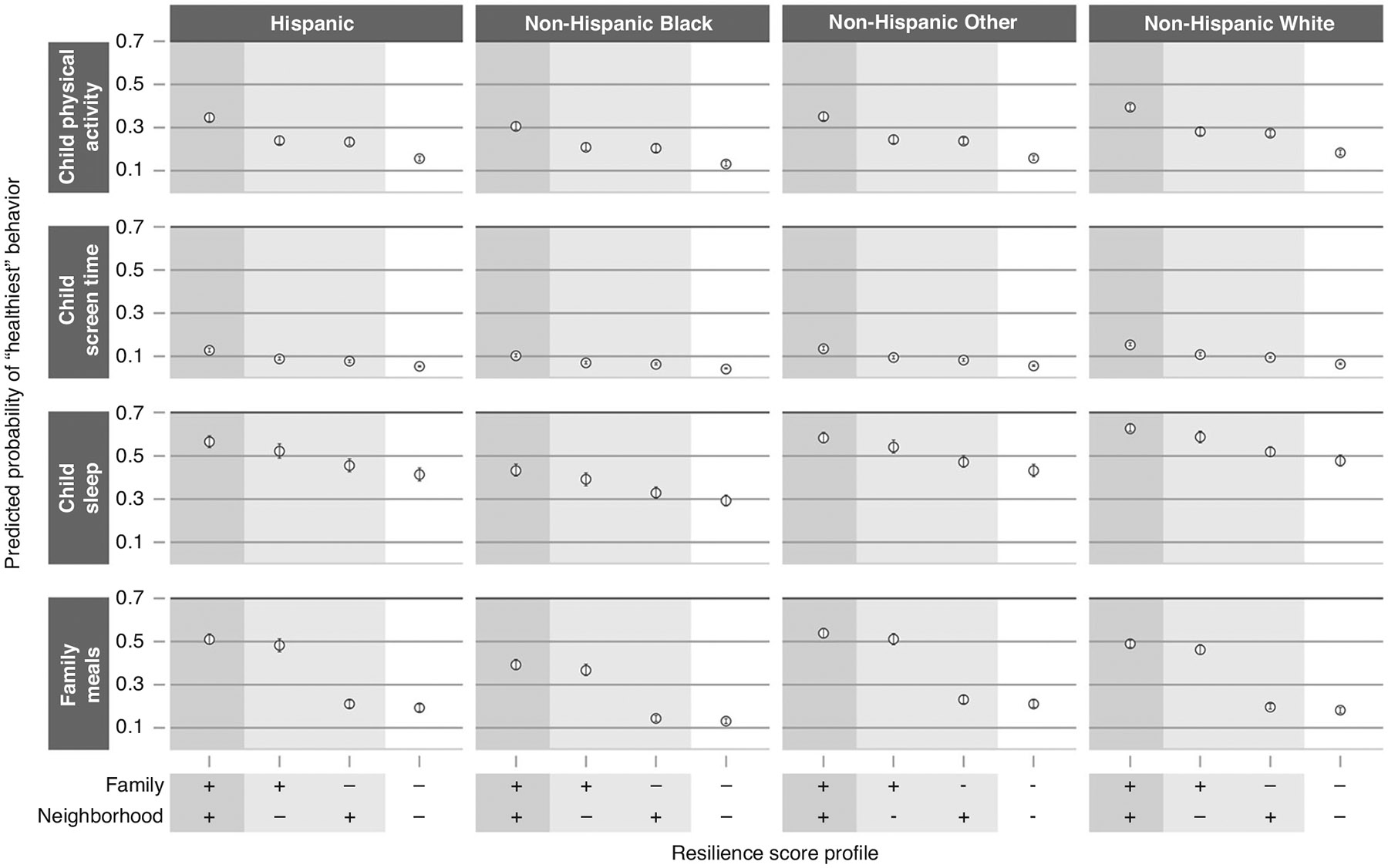
Predicted probabilities of “healthiest” behavior outcomes in 6–17 years. Results are displayed by race/ethnicity, indicated in column headings, and by health behavior outcome, indicated in headings on the left. Resilience profiles are designated along the x-axis at the bottom of the figure. For each domain (family and neighborhood), “+” indicates “high resilience” (score of 3.0) and “−” indicates “low resilience” (score of 1.5) in that domain. Covariates for all resilience profiles are set to the median or modal value. For each health behavior outcome, the y-axis represents the predicted probability of reporting the “healthiest” behavior option, with values indicated by open circles and vertical error bars representing 95% confidence intervals.

Table 3 shows odds ratios from the adjusted regression models for resilience profiles with specific combinations of high and low resilience in the age-applicable domains. For each outcome, the highest odds of better health-behavior outcomes are seen when all resilience domains are at their highest value. For example, among children ages 0–5 years, the aOR for eating family meals together more frequently was 1.07 (CI 0.97, 1.18) for high neighborhood resilience compared to low resilience in each domain, 3.35 (CI 2.94, 3.76) for high family resilience compared to low resilience in each domain, and 4.92 (CI 3.99, 5.85) for high resilience in each domain compared to low resilience in each domain. Figure 1 (0–5 year age group) and Fig. 2 (6–17 year age group) show the predicted probabilities of reporting the “best” health behavior response for each outcome for children with different resilience profiles (complete predicted probability results in Supplementary Table 2). For example, among Hispanic children ages 0–5 years, the predicted probability for eating family meals together every day was 28.0% (95% CI 24.0–32.0%) with low resilience in each domain, 30.0% (95% CI 25.0–34.0%) with high neighborhood resilience only, 57.0% (95% CI 52.0–62.0%) with high family resilience only, and 66.0% (95% CI 62.0–69.0%) with high child, family, and neighborhood resilience. The addition of interaction terms to the 6–17 year models to consider middle childhood (6–11 years) versus adolescence (12–17 years) did not significantly impact the relationship between resilience and health behaviors for any of the outcomes other than child sleep (Wald test, *p* = 0.003). The model with the additional interaction terms for the child sleep outcome by middle childhood and adolescence indicates that neighborhood resilience is more strongly associated with healthy sleep for children between 6–11 years than for those between 12–17 years. To illustrate this association using predicted probabilities, for children in the Non-Hispanic White race/ethnicity category, the predicted probability of having “good quality sleep” was the following: for both high family and high neighborhood resilience, 0.69 (0.67, 0.71) in middle childhood and 0.65 (0.63, 0.67) in adolescence; for high family resilience only, 0.62 (0.58, 0.65) in middle childhood and 0.64 (0.60, 0.67) in adolescence; for high neighborhood resilience only, 0.60 (0.57, 0.63) in middle childhood and 0.52 (0.49, 0.55) in adolescence; for neither high family nor high neighborhood resilience: 0.52 (0.49, 0.55) in middle childhood and 0.50 (0.47, 0.53) in adolescence (Supplementary Table 3 and Supplementary Fig. 1).

## DISCUSSION

Our analysis of multiple years of National Survey of Children’s Health found that resilience in the child, family, and neighborhood domains are each positively associated with multiple child and family health behavior outcomes in children ages 0–17 years, even after adjusting for the other resilience domain(s) and all covariates. When analyzing varying combinations of high and low resilience domains, we found the highest likelihood of “better” health behaviors in profiles with all age-applicable resilience domains at their highest values.

Prior studies have described resilience-promoting factors within each of the three domains of resilience measured in this study. Individual characteristics associated with a child’s resilience include having an agreeable temperament, positive self-concept, ability to regulate emotions, and adaptive and social problemsolving skills.^52,59-62^ At the family level, resilience-promoting factors include positive relationships between family members, healthy attachment and communication, certain parenting styles, and living in a household whose members are in good mental and physical health.^33,63,64^ Finally, there are resilience-promoting factors within the larger social and environmental context, such as the school, neighborhood, or community; these can include both aspects of the built environment, as well as of the social environment, such as social support, inclusion, opportunity, access to amenities, and perceived safety.^32,33,65^ Previous research has revealed associations between resilience and health behaviors in adults, as well as in limited subpopulations and resilience domains in children and adolescents^31-33,40-43,47-49,63-65^ Our study advances what is known about this field by demonstrating, in a general population of 0–17-year-old children, that multiple socio-ecological domains of resilience have individual and combined associations with positive health behaviors.

A main implication of our study results is the importance of a coordinated multi-domain approach that fosters the integration of resilience promotion across child developmental stages and between individual, family, and social/environmental factors. Research has shown that the development of protective health behaviors in childhood helps to establish a trajectory that reduces the risk of disease throughout the lifespan.^44,46,50,51^ Studies that elucidate the relationships between individual, family-level, and neighborhood-level resilience and child health behaviors may help to guide efforts to support positive health behaviors in children at risk of adverse health outcomes due to early life stress. Prior research on targeted resilience-building interventions in children has shown them to be capable both of building resilience and improving mental health outcomes.^45^ Further research should focus on designing interventions that target all three levels of resilience and evaluating intervention effectiveness and costeffectiveness from a public health standpoint, as well as how to best implement such interventions in order to reach the target populations. Additionally, research is needed to understand any differential effects of resilience on populations by socioeconomic status (SES), race/ethnicity, culture, sex, and gender.

Disparities in health behaviors and outcomes by race/ethnicity and SES highlight the need for supportive behavioral and public health interventions in systemically disadvantaged populations.^33,36,66-68^ Our study suggests that resilience may be an important intervention target to reduce these disparities given its association with multiple health behaviors in children. Furthermore, the increased likelihood of positive health behaviors seen with combined high resilience in multiple domains imply that interventions aimed at improving health behavior disparities should be multi-level and target not only individual children and their families, but also their surrounding social and structural environments that pose barriers to participation in positive health behaviors most commonly for marginalized, low-SES, and racial/ethnic minority populations.

There are several limitations to our study. As with all cross-sectional studies, our analysis of the cross-sectional NSCH data allows us to uncover associations between resilience and health behaviors but cannot prove causality or show temporality. Additionally, because the survey is conducted in only English and Spanish, we are unable to generalize our findings beyond children with English or Spanish-speaking parents. The measures of child, family, and neighborhood resilience used for this study are not validated measures, but their legitimacy is strengthened by the apparent face validity of the included survey items, which are consistent in content with described resilience constructs and validated resilience scales.^25,69,70^ Further, though individual-level resilience is conceptually important for all ages,^26^ the NSCH only collected the child resilience measure in the 0–5 year age group, limiting our analysis in the 6–17 year age group to the family and neighborhood domains. Data collected via this survey are exclusively based on parent report, which raises concern for social desirability bias, recall bias, and overall accuracy of information, especially for older children and teenagers. The sample also exhibits high median levels of resilience across domains and ages, which may not represent the general U.S. population.

This analysis of NSCH data from 2016 to 2021 of children ages 0–17 years shows associations between child, family, and neighborhood resilience and a range of positive childhood health behaviors. Furthermore, having high resilience in all relevant domains is associated with the greatest likelihood of exhibiting more positive health behaviors. Given known disparities in health behaviors that contribute to downstream health disparities, further research should focus on elucidating the direction of causality and magnitude of effect between each resilience domain and health behavior, particularly in low-income and marginalized racial and ethnic populations. The long-term goal of this line of research is to develop effective multilevel resilience-building interventions that improve health behaviors and thus decrease disparities in poor health outcomes.

## Supplementary Material

Supplemental Material

Supplemental Table

## Figures and Tables

**Table 1.** Demographics in weighted sample.

Characteristics	Ages 0–5 Years *N* = 22,526,805	Ages 6–17 Years *N* = 47,629,735
Child age (years), median (IQR^a^)	3.00 (1.00, 4.00)	12.0 (9.0, 14.0)
Child sex, *n*(%)		
Male	11,498,993 (51%)	24,335,941 (51%)
Female	11,027,812 (49%)	23,293,793 (49%)
Child race/ethnicity category, *n*(%)		
Hispanic	5,396,235 (24%)	12,156,786 (26%)
Non-Hispanic Black	2,606,273 (12%)	6,302,217 (13%)
Non-Hispanic Other	2,700,354 (12%)	4,861,139 (10%)
Non-Hispanic White	11,653,479 (52%)	23,965,762 (51%)
Unknown, *n*	170,463	343,830
Primary household language, *n*(%)		
English	19,207,272 (86%)	40,453,934 (86%)
Spanish	1,882,470 (8.4%)	4,947,982 (10%)
Other	1,297,428 (5.8%)	1,838,827 (3.9%)
Unknown, *n*	139,634	388,992
Highest education among household adults, *n*(%)		
Less than high school	1,460,003 (6.5%)	4,805,806 (10%)
High school	3,970,678 (18%)	9,360,931 (20%)
Some college or associate degree	4,720,610 (21%)	10,315,869 (22%)
College degree or higher	12,281,941 (55%)	22,977,184 (48%)
Unknown, *n*	93,574	169,944
Family Poverty Ratio^b^, *n*(%)		
≤50–99%	3,038,457 (16%)	6,293,451 (16%)
100–199%	3,715,443 (20%)	8,088,349 (21%)
200–399%	5,473,987 (30%)	11,396,584 (29%)
≥400%	6,327,958 (34%)	12,954,251 (33%)
Unknown, *n*	3,970,960	8,897,100
Special health care needs in child		
Yes	2,348,753 (10%)	11,089,800 (23%)
No	20,178,052 (90%)	36,539,935 (77%)
Child ACEs^c^ category		
0 ACES	10,584,461 (49%)	15,278,357 (34%)
1–3 ACES	10,274,252 (48%)	25,568,789 (57%)
4–9 ACES	536,967 (2.5%)	3,860,274 (8.6%)
Unknown, *n*	1,131,126	2,922,315

a *IQR* interquartile range.

b Calculated as the ratio of total family income to the family poverty threshold (derived from the Census Bureau’s poverty thresholds) and reported as a rounded percentage. Reported values range from 50 (total family income is 50% of the family poverty threshold) to 400 (total family income is 400% of the family poverty threshold).

c *ACEs* adverse childhood experiences.

**Table 2.** Observed resilience profile distributions in weighted sample.

Resilience profile^a^	Ages 0–5 Years *N* (%)	Ages 6–17 Years *N* (%)
*C* + *F* + *N*	7,091,192 (31.5%)	N/A^b^
*C* + *F*	1,294,056 (5.7%)	N/A
*C* + *N*	211,386 (0.9%)	N/A
*F* + *N*	71,187 (0.3%)	14,839,753 (31.2%)
*C*	300,563 (1.3%)	N/A
*F*	39,198 (0.2%)	1,519,621 (3.2%)
*N*	16,587 (0.1%)	1,167,752 (2.5%)
*None (all low)*	21,924 (0.1%)	811,315 (1.7%)
*Not meeting profile criteria* ^c^	13,480,713 (59.5%)	29,291,293 (61.5%)

a Resilience profiles are labeled according to which domains have “high” resilience: *C* = high child resilience, *F* = high family resilience, and *N* = high neighborhood resilience. Thus, “*C* + *F* + *N*” denotes high resilience in all three domains. We categorized “high” resilience scores as 2.5–3.0 and “low” resilience scores as 0–1.5.

b *N/A* not applicable. This is due to the NSCH survey methodology; specifically, the survey items used in our study to assess the child resilience domain were administered only to the 0–5 year age group but not the 6–17 year age group.

c Observations in any domain with a score between 1.5 and 2.5 do not meet criteria for the listed resilience profiles.

**Table 3.** Adjusted odds ratios of better health behaviors by resilience profile^a^.

*Ages 0–5 years*							
Resilience profile^b^							
Health Behavior	*C + F + N aOR*^c^ *(95%CI*^d^)	*C + F aOR (95%CI)*	*C + N aOR (95%CI)*	*F + N aOR (95%CI)*	*C aOR (95%CI)*	*F aOR (95%CI)*	*N aOR (95%CI)*
*Screen Time*	1.96 (1.62, 2.29)	1.44 (1.20, 1.68)	1.41 (1.17, 1.64)	1.89 (1.65, 2.12)	1.04 (0.88, 1.19)	1.39 (1.24, 1.54)	1.36 (1.23, 1.48)
*Sleep Quality*	2.21 (1.78, 2.63)	1.94 (1.59, 2.29)	1.69 (1.38, 2.00)	1.49 (1.27, 1.70)	1.49 (1.26, 1.72)	1.31 (1.15, 1.46)	1.14 (1.01, 1.26)
*Family Meals*	4.92 (3.99, 5.85)	4.59 (3.78, 5.41)	1.47 (1.22, 1.72)	3.59 (3.06, 4.12)	1.37 (1.17, 1.57)	3.35 (2.94, 3.76)	1.07 (0.97, 1.18)
*Reading*	2.66 (2.20, 3.11)	2.35 (1.97, 2.74)	1.66 (1.38, 1.94)	1.82 (1.58, 2.05)	1.47 (1.24, 1.69)	1.61 (1.44, 1.77)	1.13 (1.03, 1.23)
*Singing/Storytelling*	3.73 (3.08, 4.38)	3.57 (2.97, 4.17)	1.95 (1.63, 2.28)	2.00 (1.73, 2.27)	1.87 (1.59, 2.15)	1.91 (1.70, 2.12)	1.05 (0.95, 1.15)
*Ages 6–17 Years*							
Resilience Profile							
Health Behavior	N/A^e^	N/A	N/A	*F + N aOR (95%CI)*	N/A	*F aOR (95%CI)*	*N aOR (95%CI)*
*Physical Activity*	N/A	N/A	N/A	2.88 (2.62, 3.14)	N/A	1.73 (1.61, 1.85)	1.66 (1.53, 1.79)
*Screen Time*	N/A	N/A	N/A	2.60 (2.38, 2.82)	N/A	1.73 (1.62, 1.84)	1.50 (1.39, 1.62)
*Sleep Quality*	N/A	N/A	N/A	1.85 (1.67, 2.03)	N/A	1.56 (1.44, 1.67)	1.19 (1.08, 1.30)
*Family Meals*	N/A	N/A	N/A	4.36 (3.96, 4.76)	N/A	3.90 (3.62, 4.18)	1.12 (1.03, 1.21)

a Each row represents output from a separate model where all measured resilience domains are included while also adjusting for other covariates. All results are with reference to a profile with low resilience in all age-applicable domains. Results give the odds of engaging in a “better” vs. “worse” health behavior response option; namely, more frequent physical activity, less daily screen time, good as opposed to poor sleep quality, more frequent family meals, more frequent reading, more frequent singing/storytelling.

b Resilience profiles are labeled according to which domains have “high” resilience, where “high” resilience is a score of 3.0 and “low” resilience is a score of 1.5. *C* = high child, *F* = high family, and *N* = high neighborhood resilience. Thus, *“C + F + N”* denotes high resilience in all three domains.

c *aOR* adjusted odds ratio.

d *95%CI* 95% confidence interval.

e *N/A* not applicable.

## Data Availability

The National Survey of Children’s Health data used in this study is publicly available through the United States Census Bureau at [https://www.census.gov/programssurveys/nsch/data/datasets.html] and the U.S. Department of Health and Human Services, Health Resources and Services Administration (HRSA), Maternal and Child Health Bureau (MCHB) at [www.childhealthdata.org].

## IMPACT:

- This study assessed the associations between three socio-ecological resilience domains (child, family, and neighborhood) and six child and family health behaviors in a national dataset.
- Resilience exists within multiple socio-ecological levels and supports healthy functioning despite experiencing stress. Studies in adults and limited pediatric sub-populations show associations between resilience and health behaviors, which in turn influence numerous health outcomes.
- Resilience at three levels of socio-ecological levels was found to be associated with the performance of multiple child and family health behaviors in a nationally representative general pediatric population. These findings have important implications for child and family health promotion efforts.
