# Supplementary material for: The relationships between resilience and child health behaviors in a national dataset
Source: Pediatr Res. 2024 Oct 21;97(7):2296–304. doi: 10.1038/s41390-024-03664-9 (PMC12039962; doi:10.1038/s41390-024-03664-9)
Supplement: Supplementary file 1 — Supplemental Material [file 41390_2024_3664_MOESM1_ESM.pdf]

Supplemental Table 1. Exposure and Outcome Distribution in Weighted Sample

**Characteristic**	**Ages 0-5 years**<br>N = 22,526,805<br>n(%) unless otherwise noted	**Ages 6-17 years**<br>N = 47,629,735<br>n(%) unless otherwise noted
Exposures[a]		
Child Resilience Score, median (IQR[b])	2.75 (2.50, 3.00)	N/A[c]
Unknown, n	281,566	N/A
Family Resilience Score, median (IQR)	2.75 (2.00, 3.00)	2.50 (2.00, 3.00)
Unknown, n	432,853	973,855
Neighborhood Resilience Score, median (IQR)	2.50 (2.00, 3.00)	2.40 (2.00, 2.80)
Unknown, n	496,994	1,317,305
Outcomes		
Child Physical Activity (Weekly)		
0 days	N/A	4,869,754 (10%)
1-3 days	N/A	19,389,172 (41%)
4-6 days	N/A	12,927,483 (27%)
Every day	N/A	10,443,326 (22%)
Child Screen Time (Daily)		
4 or more hours	2,495,853 (11%)	16,136,476 (34%)
3 hours	2,735,956 (12%)	9,416,620 (20%)
2 hours	5,380,304 (24%)	12,358,950 (26%)
1 hour	4,330,242 (19%)	6,066,300 (13%)
Less than 1 hour	7,584,449 (34%)	3,651,388 (7.7%)
Child Sleep Quality (Daily)		
Poor sleep quality	8,886,233 (39%)	26,449,212 (56%)
Good sleep quality	13,640,573 (61%)	21,180,523 (44%)
Family Meals Together (Weekly)		
0 days	464,251 (2.1%)	1,736,883 (3.6%)
1-3 days	3,653,042 (16%)	12,378,301 (26%)
4-6 days	5,791,332 (26%)	14,477,970 (30%)
Every day	12,618,180 (56%)	19,036,581 (40%)
Time Reading to Child (Weekly)		
0 days	1,727,282 (7.7%)	N/A
1-3 days	8,100,651 (36%)	N/A
4-6 days	4,246,098 (19%)	N/A
Every day	8,452,774 (38%)	N/A
Time Singing/Storytelling to Child (Weekly)		
0 days	911,442 (4.0%)	N/A
1-3 days	6,332,878 (28%)	N/A
4-6 days	4,253,679 (19%)	N/A
Every day	11,028,806 (49%)	N/A

a. For all exposure variables, the possible score range is 0-3 with 3 indicating the highest possible resilience in that domain.
b. IQR = interquartile range
c. N/A = not applicable

Supplemental Table 2. Multiple Combined Domain Results: Predicted Probabilities of "Best" Health Behavior Outcome

Please find Supplemental Table 2 in the accompanying excel file.

Supplemental Table 3. Multiple Combined Domain Results: Predicted Probabilities of “Good Quality Sleep” in Middle Childhood and Adolescence

Ages 6-11 Years (“Middle Childhood”)				
**Health Behavior Outcome**				
**Resilience Profile**[a]	**Race/Ethnicity**			
	Hispanic	*Non-Hispanic Black*	*Non-Hispanic Other*	*Non-Hispanic White*
F + N	0.63 (0.60, 0.66)	0.50 (0.47, 0.54)	0.65 (0.62, 0.68)	0.69 (0.67, 0.71)
F	0.55 (0.51, 0.59)	0.42 (0.38, 0.46)	0.57 (0.54, 0.61)	0.62 (0.58, 0.65)
N	0.54 (0.50, 0.57)	0.41 (0.37, 0.44)	0.56 (0.52, 0.59)	0.60 (0.57, 0.63)
None	0.45 (0.42, 0.49)	0.33 (0.29, 0.36)	0.47 (0.44, 0.51)	0.52 (0.49, 0.55)
Ages 12-17 Years (“Adolescence”)				
	Hispanic	*Non-Hispanic Black*	*Non-Hispanic Other*	*Non-Hispanic White*
F + N	0.59 (0.56, 0.62)	0.46 (0.42, 0.49)	0.61 (0.58, 0.64)	0.65 (0.63, 0.67)
F	0.57 (0.53, 0.61)	0.44 (0.40, 0.48)	0.59 (0.55, 0.63)	0.64 (0.60, 0.67)
N	0.45 (0.41, 0.49)	0.33 (0.29, 0.36)	0.47 (0.44, 0.51)	0.52 (0.49, 0.55)
None	0.43 (0.40, 0.47)	0.31 (0.28, 0.34)	0.45 (0.42, 0.49)	0.50 0.47, 0.53)

a. Resilience profiles are labeled according to which domains have “high” resilience: *F* = high family resilience, and *N* = high neighborhood resilience. Thus, “*F + N*” denotes high resilience in both domains. We categorized “high” resilience scores as 2.5-3.0 and “low” resilience scores as 0-1.5.

Supplemental Figure 1. Predicted Probabilities of “Good Quality Sleep” in Middle Childhood and Adolescence

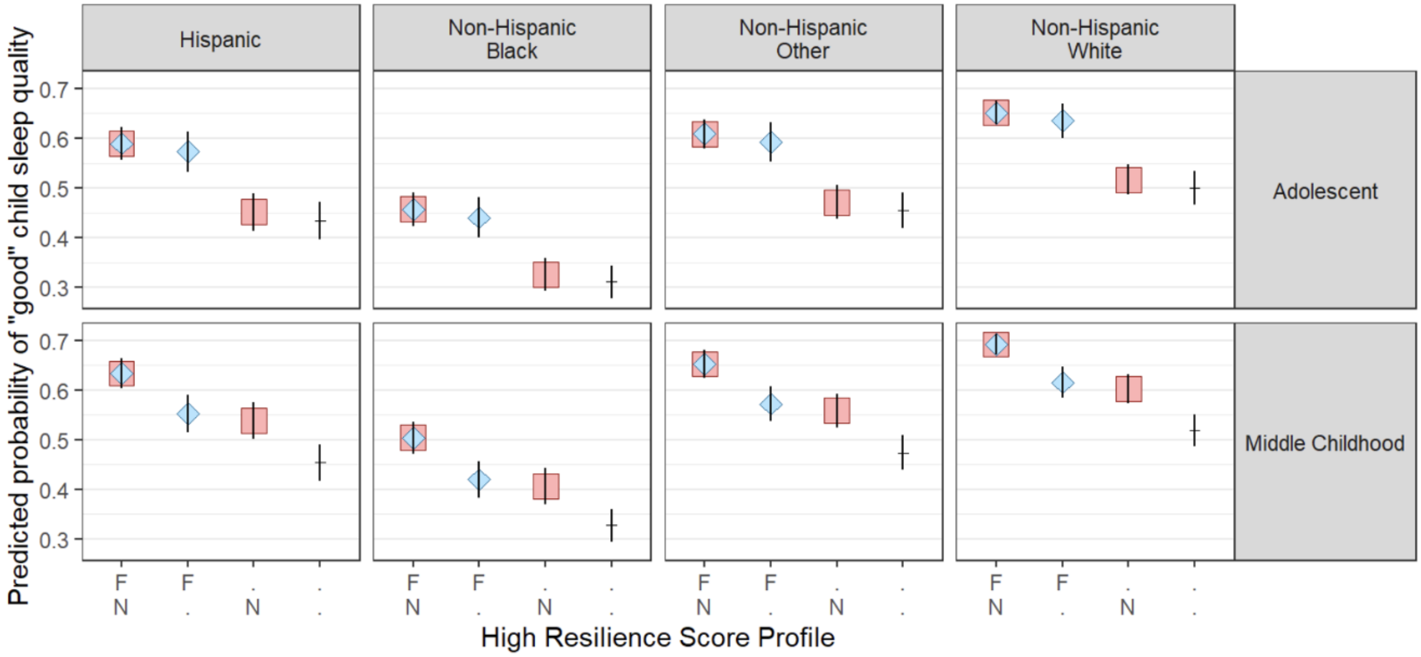

NOTE: “Middle Childhood” includes ages 6-11 years. “Adolescent” includes ages 12-17 years. Resilience profiles are on the x-axis of the bottom row and are labeled according to which domains have “high” resilience: *F* = high family resilience, and *N* = high neighborhood resilience, such that “high” resilience score is set at 3.0 and compared to a “low” resilience score at 1.5. Thus, “*F* + *N*” denotes high resilience in both domains. The y-axis represents the predicted probabilities of reporting the “best” health behavior option, that is, “good quality sleep,” Predicted probabilities for each profile are indicated by a *red square* for high neighborhood resilience, a *blue diamond* for high family resilience, and a *horizontal line* for low neighborhood and family resilience. Vertical error bars represent the 95% confidence intervals. For all resilience profiles, covariates are set to the median or modal value in the 6-17 age group except for race/ethnicity.

Appendix: Survey Measurement Details

Exposure Survey Items

Child resilience was measured among children ages 0-5 years using four survey items: "How often…" (1) is this child affectionate and tender with you? (2) does this child bounce back quickly when things do not go their way? (3) does this child show interest and curiosity in learning new things? (4) does this child smile and laugh? Response options were "always", "usually", "sometimes", or "never," coded from 0-"never" to 3-"always," such that scores ranged from 0-12.

Family resilience was measured among children ages 0-17 years with four survey items: "When your family faces problems, how often are you likely to do each of the following?" (1) Talk together about what to do, (2) Work together to solve our problems, (3) Know we have strengths to draw on, and (4) Stay hopeful even in difficult times. Response options were "none of the time," "some of the time," "most of the time," or "all of the time," coded from 0-"none of the time" to 3-" all of the time," such that scores ranged from 0-12.

Neighborhood resilience was measured in among children ages 0-17 years with four survey items: "To what extent do you agree with these statements about your neighborhood or community?" (1) People in this neighborhood help each other out, (2) We watch out for each other's children in this neighborhood, (3) This child is safe in our neighborhood, and (4) When we encounter difficulties, we know where to go for help in our community. Households with children aged 6-17 years were asked a fifth survey item: (5) This child is safe at school. Response options were "definitely disagree," "somewhat disagree," "somewhat agree," or "definitely agree," coded from 0-"definitely disagree" to 3-" definitely agree," such that scores ranged from 0-12 for ages 0-5 years and from 0-15 for ages 6-17 years.

For all resilience domains, we averaged responses across the survey items within each domain to create continuous measures of resilience (range: 0-3) for each child at each age-applicable level.

Outcome Measurement Details

Physical activity was measured in children ages 6-17 years with the item, "During the past week, on how many days did this child exercise, play a sport, or participate in physical activity for at least 60 minutes?" Response options were "0 days," "1-3 days," "4-6 days," or "every day," coded from 1-"0 days" to 4-"every day".

Child screen time was measured in children ages 0-17 years with two items in the survey administered in 2016-17, and one item in 2018-21. In 2016-17, the items read, (1) "On an average weekday, about how much time does [your child] usually spend in front of a TV watching TV programs, videos, or playing video games?" and (2) "On an average weekday, about how much time does [your child] usually spend with computers, cell phones, handheld video games, and other electronic devices, doing things other than schoolwork?" Response options were "None", "Less than 1 hour," "1 hour," "2 hours," "3 hours," or "4 or more hours." We added the reported hours from these two questions to get a total number of average daily hours of screen time per child. Responses of "None" were combined with "Less than 1 hour." In 2018-21 the single item read, "On most weekdays, about how much time does this child usually spend in front of a TV, computer, cell phone, or other electronic device watching programs, playing games, accessing the internet, or using social media? Do not include time spent doing schoolwork." Response options were "Less than 1 hour," "1 hour," "2 hours," "3 hours," or "4 or more hours." The derived 2016-21 screen time measure was coded on a 5-point Likert scale from 1-"Less than 1 hour" to 5-"4 or more hours," and then reverse-coded from 1-"4 or more hours" to 5-"Less than 1 hour" for consistency with other outcome measures in which higher values represent better health behavior outcomes (in this case, less screen time).

Child sleep quality was assessed in children ages 0-17 via two sleep characteristics: (1) bedtime consistency and (2) average daily sleep amount. Bedtime consistency was measured among children ages 0-17 with the single survey item, "How often does this child go to bed at about the same time on weeknights?" Response options were: "Always," "Usually," "Sometimes," "Rarely," and "Never", dichotomized such that responses of "Always" or "Usually" indicate "regular bedtime" and responses of "Rarely" or "Never" indicate "irregular bedtime". Sleep amount was measured among children ages 0-5 with the survey item, "During the past week, how many hours of sleep did this child get during an average day? Count both nighttime and sleep naps." Response options were "Less than 7 hours," "7 hours," "8 hours," "9 hours," "10 hours, "11 hours," or "12 or more hours." Among children ages 6-17, the item read: "During the past week, how many hours of sleep did this child get on most weeknights?" Response options were "Less than 6 hours," "6 hours," "7 hours," "8 hours," "9 hours," "10 hours," or "11 or more hours." Hours of sleep were dichotomized such that responses that meet the American Academy of Sleep Medicine's recommended range of hours for the child's continuous age designate "adequate sleep"; if otherwise, "inadequate sleep". The derived child sleep quality outcome

dichotomizes children meeting the criteria for both “routine bedtime” and “adequate sleep” as having “good sleep quality”; if otherwise, “poor sleep quality.”

Frequency of eating meals together as a family was measured in children ages 0-17 years with the item, “During the past week, on how many days did all the family members who live in the household eat a meal together?” Response options were “0 days,” “1-3 days,” “4-6 days,” or “every day,” coded from 1-“0 days” to 4-“every day”.

Household literacy-promoting behaviors were measured in ages 0-5 years with two items: (1) “During the past week, how many days did you or other family members tell stories or sing songs to this child?” and (2) “During the past week, how many days did you or other family members read to this child?” Response options for both were “0 days,” “1-3 days,” “4-6 days,” or “every day,” coded from 1-“0 days” to 4-“every day”.

Covariate Measurement Details

Exposures to adverse childhood experiences (ACEs) were measured via nine binary items asking if the child had ever experienced the following: 1) parent or guardian divorced or separated, 2) parent or guardian died, 3) parent or guardian jail time, 4) witnessed domestic violence, 5) witnessed or was the victim or neighborhood violence, 6) household mental illness, 7) household alcohol or drug abuse, 8) treated unfairly because of race or ethnic group, or 9) difficulty covering basics like food or housing. Response options for each were “yes” or “no.” The number of "yes" responses was summed across these items for a total possible range of 0-9 ACEs per child and then categorized by count into the following possible ranges for analysis: 0, 1-3, and 4-9 ACEs.
